# A Friendly Cross-Examination: By a Special Correspondent

**Published:** 1920-05-22

**Authors:** 


### THE HOSPITAL SATURDAY FUND.

### A Friendly Cross-examination: By a Special Correspondent.

Under this title there appeared last week in The Hospital an admirable letter from Mr. Philip A. Inman, the secretary of the Metropolitan Hospital Saturday Fund. His object was to record briefly the activities of the fund at the present time in order to remove any "erroneous impression" which might be left by the statement in The Hospital of May 1:—"We fear that the Fund touches but a very small percentage of the works and workshops of London." This statement has led Mr. Inman very properly to put on record the figures of the work done by the Fund, and a statistical summary of its activities. The figures are so instructive that I propose to examine them at leisure, and in the order mentioned by Mr. Inman.

### Where Collections are Made.

"Collections," he says, "are made week by week in 20,000 Metropolitan workshops, factories and business houses, and it is estimated that 400,000 people are regular subscribers to these collections." It requires a great deal of very hard work to organise 20,000 collections anywhere, but whether 20,000 collections is a satisfactory number or not depends upon two things. First, the number of workshops, factories and business houses in the area from which collections might be made, and secondly, on the sum realised from each collection. Mr. Inman's careful phrase is yet comprehensive. He speaks of "workshops, factories and business houses." Is 20,000 a satisfactory proportion of all the places in London which can reasonably be included in this description? Does it include, for instance, the banks, offices, all those places which the suburban trains hurry to fill in the morning and hasten to empty before night? Does it include all retail shops of any pretension in the four-mile radius? If these are included in the definition, can it be fairly denied that 20,000 is a small proportion of the whole? Mr. Inman is fully alive to "opportunities for advancement," and like an active man is more concerned with these than with the past. Perhaps, then, he has estimated the number of workshops, factories and business houses in London which the Hospital Saturday Fund *has yet to touch*. The publication of such a table would in no sense cast a shadow on existing achievements, rather it would encourage every sympathiser to surpass them by defining the amount of work ahead. To find the correct figure a precise allowance would have to be made for "the large number of firms" whose employees contribute direct to their local hospital." What is this large number? Does anyone exactly know? Can Mr. Inman add to his valuable statement the correct figure? We are all agreed that, at the present crisis, those who support the voluntary system need most to know the precise extent of the public loaf which remains at present *un*leavened. We must all take stock, and for this reason Mr. Inman's figures already published are most valuable.

We now pass from the number of the collections, and the question whether 20,000 represents a satisfactory proportion of collecting centres, to the sum total which these collections raise. Mr. Inman says "it is estimated that 400,000 people are regular subscribers to these collections." What does arithmetic tell us about this? It tells us that the average number of subscribers to each collection is twenty. The figure is interesting because it suggests the size of the average workshop, factory, or business house in which a collection is arranged. Of course, in some centres, perhaps in most, the number of subscribers will be immensely greater. That which we need to know is the smallest number of persons from which it is thought worth while to organise a collection at present.

The number of honorary workers is mentioned too broadly for any inference to be based upon it, beyond a sense of sincere gratitude to them and to their work.

The next precise figure is that of "the thirty-three strong and active local committees working enthusiastically in and around the city of London." Whether this is a satisfactory number, and how far the grouping is strategic, a map alone can show. If Mr. Inman has one, and would publish it, the map would be a most instructive study.

### An Important Figure.

The next important figure is that of the income of the fund for 1919, which is recorded to have been a sum of £74,848. Call this £75,000 and divide it by the "400,000 regular subscribers" mentioned above. It would appear, therefore, that each subscriber, assuming the income to be derived entirely from subscriptions, gives to the fund a sum of 3s. 9d. a year, or less than one penny a week. Is this a satisfactory subscription? The direct subscriptions to a local hospital do not affect this result, which is independent of them.

In regard to the 50,000 spectators present at the Cup-tie football match at Stamford Bridge, Mr. Inman, very justly, points out that many of them came from the provinces. This fact, however, suggests that they might have been invited to subscribe for the occasion. Cannot collections be arranged upon the ground at the great matches? Need "the supporters of the Chelsea Football Club" be the sole or chief group of footballers provided with an opportunity to contribute? Could not the privilege be extended to others?

The object of this analysis is not to embarrass Mr. Inman in a heavy task, but to encourage Londoners under his leadership to make the most of the "opportunities for advancement" which he mentions. A definition of the work yet to be done is the first step towards its accomplishment. It is the duty of us all to know exactly where we are upon the steep hill which must be climbed if the Metropolitan Hospital Saturday Fund is to make the most of its enormous opportunity. Lastly, the population of London, within the purview of the

**The Hospital Saturday Fund**—(*continued*).

fund, can hardly be put lower than two millions. Of this multitude an uncertain fraction subscribes direct to its local hospital, and 400,000 are regular subscribers to the fund. Is this a satisfactory proportion? There can be only one answer. Of the twenty possible persons, four only are regular subscribers. It is the remaining sixteen whom the fund has to get. When they shall have been gained, even on the present basis of subscription, the income of the fund will be two million three-shilling-and-ninepences, or, in other words, £375,000. Reduce this by half, as we may do when reducing theory to practice, and we arrive at an income of £187,500. That is the income which a sober judgment can reasonably expect from the Metropolitan Hospital Saturday Fund. It would be trifling with the whole question to be content with a sovereign less than that. Presumably plans are afoot to raise it by the end of the year.

